# Peptide targeting the interaction of S protein cysteine-rich domain with Ezrin restricts pan-coronavirus infection

**DOI:** 10.1038/s41392-022-01244-z

**Published:** 2023-01-18

**Authors:** Zhuanchang Wu, Xiaobo Lei, Xin Wang, Zhaoying Zhang, Yuming Li, Lifen Gao, Xiaohong Liang, Peihui Wang, Jianwei Wang, Chunhong Ma

**Affiliations:** 1 Key Laboratory for Experimental Teratology of Ministry of Education, Key Laboratory of Infection and Immunity of Shandong Province and Dept. Immunology, School of Basic Medical Sciences, Cheeloo Medical College, Shandong University, 250012 Jinan, Shandong China; 2 NHC Key Laboratory of Systems Biology of Pathogens, Institute of Pathogen Biology, Chinese Academy of Medical Sciences and Peking Union Medical College, 100730 Beijing, China; 3 College of Agriculture and Forestry, Linyi University, Linyi, Shandong China; 4 School of Public Health, Shandong First Medical University & Shandong Academy of Medical Sciences, 271016 Tai’an, China; 5 Key Laboratory for Experimental Teratology of Ministry of Education and Advanced Medical Research Institute, Shandong University, 250012 Jinan, Shandong China

**Keywords:** Microbiology, Drug development

**Dear Editor**,

To date, seven human coronaviruses (HCoVs) have been identified, among which the highly pathogenic severe acute respiratory syndrome-associated coronavirus (SARS-CoV), Middle East respiratory syndrome coronavirus (MERS-CoV), and severe acute respiratory syndrome coronavirus 2 (SARS-CoV-2) have caused public health disasters worldwide. More importantly, currently available vaccines and antibodies become less effective in neutralizing the recently emerging SARS-CoV-2 immune-escape variants.^1^ Thus, it is urgent to develop universal coronavirus drugs against the emerging or the next coronavirus pandemic. Coronaviruses enter host cells via spike (S) glycoprotein, which mainly comprises two functional subunits S1 and S2 responsible for attachment and membrane fusion, respectively. The S2 subunit contains a hydrophobic fusion peptide (FP), two heptad repeat regions (HR1/HR2), and a cytoplasmic C-terminal domain (CTD). HR1/HR2 are relatively conserved among HCoVs and peptide targeting HR1 domain has exhibited an effective inhibitory activity against multiple HCoVs.^2^ CTD is only about 40 amino acids (aa) and contains a cysteine-rich domain (CRD). We recently reported that palmitoylation of S-CRD contributes to S protein-mediated cell fusion and SARS-CoV-2 pseudovirus infection.^3^ However, whether CRD serves as a target for the development of pan-CoV inhibitor remains unclear.

We first compared the cytoplasmic sequences of S proteins from representative strains of *Coronavirinae* subfamily comprising α-, β-, γ-, and δ-coronavirus genera. As shown in Supplementary Fig. S1a, b, CRD domain was found in all *Coronavirinae* subfamily members but not in its relative *Toroviridae* subfamily of *Coronaviridae*. S-CRD is comprised of about 20 aa containing 6–10 interspaced cysteine residues and the arrangement of these cysteines showed an evolutionary homology among different *Coronavirinae* members (Supplementary Fig. S1a). Furthermore, GISAID database analysis showed that there is no mutation in the CRD domain of all SARS-CoV-2 clades including the Beta, Delta, and Omicron variants (Supplementary Fig. S2). These data indicate that the CRD domain especially exists in S protein of *Coronavirinae* family and is highly conserved in SARS-CoV-2 variants.

To further investigate whether CRD-mediated cell–cell fusion is conserved, we introduced the substitution of cysteine with alanine residues in CRD domain (S-mCRD) of five HCoVs. Then, membrane fusion was estimated in coculture of ACE2^+^DPP4^+^APN^+^ Huh7 cells and HEK293T cells transfected with either wild-type S (S-WT)/GFP or S-mCRD/GFP. Fluorescent imaging showed that SARS-CoV-2 S-WT induced big syncytia formation, while S-mCRD expressing cells failed to induce syncytia (Fig. 1a). Similar results were also found in other HCoVs, including SARS-CoV, MERS-CoV, HCoV-229E, and HCoV-NL63 (Fig. 1a). Furthermore, pseudoviral infectivity assay^3^ found that the infectivity of all five pseudoviruses harboring S-mCRD was significantly decreased than that of S-WT controls (Fig. 1a). These data suggest that CRD is a conserved functional domain for S protein-mediated membrane fusion and infection of multiple HCoVs.

**Fig. 1:**
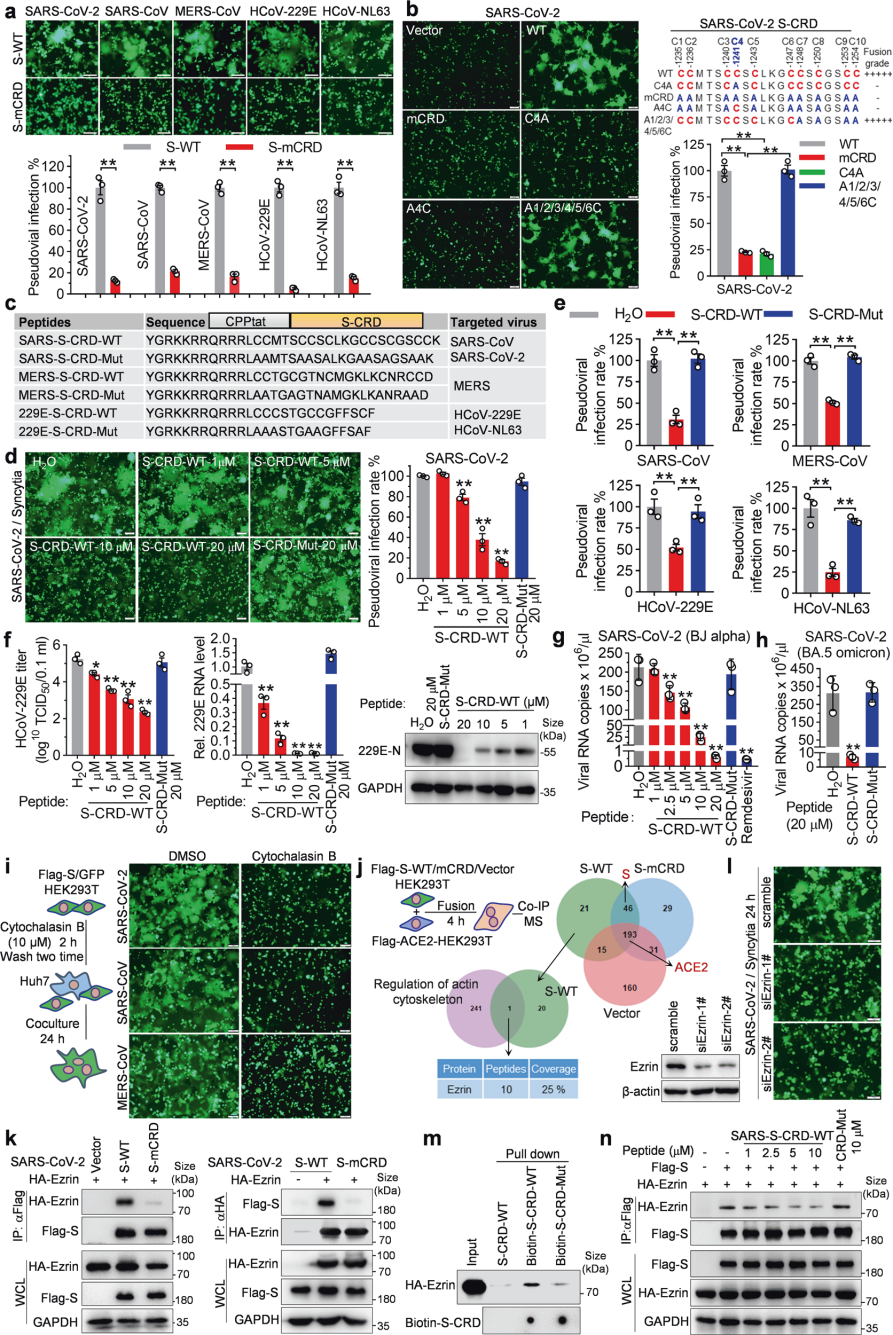
Peptide targeting the interaction of S protein CRD domain with Ezrin restricts pan-coronavirus infection. **a** HEK293T cells cotransfected with pEGFP-N1 and S-WT or S-mCRD constructs of different HCoVs were cocultured with ACE2^+^DPP4^+^APN^+^ Huh7 cells for 24 h, syncytia formation was visualized by fluorescent imaging. For SARS-CoV and HCoV-NL63 S-mediated cell fusion, trypsin (80 ng/ml) was added in DMEM without FBS to promote cell fusion. The scale bar indicates 100 µm. The pseudoviruses bearing S-WT or S-mCRD of different HCoVs infected Huh7 cells for 72 h, the firefly luciferase activity was detected to analyze viral entry (*n* = 3), which is shown in the bottom panel. Unpaired *t* test, *P < 0.05; **P < 0.01. **b** Syncytia formation induced by SARS-CoV-2 S-WT/GFP and S-CRD mutants/GFP was visualized by fluorescent imaging (left panel). Upper right panel shows the amino acid composition of CRD in S-WT and S-CRD mutants, and lower right panel shows the infectivity of pseudoviruses containing SARS-CoV-2 S-WT and S-CRD mutants detected as **a** (*n* = 3). The scale bar indicates 100 µm. One-way ANOVA, *P < 0.05; **P < 0.01. **c** Schematic, sequence, and targeted coronaviruses of S-CRD peptides. CPPtat: CPP from HIV-1 Tat protein. **d** HEK293T cells transfected with SARS-CoV-2 S-WT/GFP were treated with S-CRD peptide at indicated doses and cocultured with Huh7 cells, and syncytia formation was visualized by fluorescent imaging. The scale bar indicates 100 µm. Infectivity of SARS-CoV-2 pseudoviruses treated with S-CRD peptide was detected as **a** and shown in right panel (*n* = 3). One-way ANOVA, *P < 0.05; **P < 0.01. **e** Viral infectivity of SARS-CoV, MERS-CoV, HCoV-229E, and HCoV-NL63 pseudoviruses which was treated with S-CRD peptide (*n* = 3). One-way ANOVA, *P < 0.05; **P < 0.01. **f** Huh7 cells were infected with HCoV-229E virus at 0.1 MOI for 6 h and then treated with 229E-S-CRD peptide at the indicated doses for 36 h, viral titer and RNA levels were detected by TCID_50_ titration and RT-qPCR, respectively (*n* = 3). One-way ANOVA, *P < 0.05; **P < 0.01. N protein expression was detected by western blot. **g**, **h** Calu-3 cells were infected with 0.01 MOI SARS-CoV-2 (BJ strain of alpha variant) (**g**) or SARS-CoV-2 (BA.5 strain of omicron variant) (**h**) for 2 h and then treated with SARS-S-CRD peptide at indicated doses for 36 h (**g**), or treated with 20 μM SARS-S-CRD peptide for 36 h (**h**), viral copies in the supernatant were measured with RT-qPCR, 20 µM remdesivir-treated cell was taken as a positive control (*n* = 3). One-way ANOVA, *P < 0.05; **P < 0.01. **i** HEK293T cells transfected with SARS-CoV-2, SARS-CoV, or MERS-CoV S-WT/GFP were treated with 10 μM cytochalasin B for 2 h, and then cocultured with Huh7 cells for 24 h, syncytia formation was visualized by fluorescent imaging. The scale bar indicates 100 µm. **j** SARS-CoV-2 S-WT, S-mCRD, and vector-transfected HEK293T cells were cocultured with Flag-ACE2-HEK293T cells for 4 h, and Co-IP assay was performed with anti-Flag antibody. S-interacted proteins were screened by MS, and Venn analysis was performed. ACE2 identified by MS in all three groups indicates the validation of Co-IP and MS assay. **k** Co-IP assay was performed to analyze the interaction of HA-Ezrin with SARS-CoV-2 S-WT or S-CRD. **l** HEK293T cells were transfected with Ezrin-siRNA for 36 h and S-WT/GFP for another 36 h, and cell fusion was analyzed via coculturing with Huh7 cells. The scale bar indicates 100 µm. The knockdown effect of Ezrin was verified by western blot. **m** Biotin-S-CRD-WT peptide was incubated with cell lysate of HEK293T cells over-expressing HA-Ezrin. Pull-down assay was performed with streptavidin-beads and the pulled down proteins were detected by western blot and dot blot, Biotin-S-CRD-Mut peptide and unlabeled S-CRD-WT peptide taken as control. **n** Flag-S/HA-Ezrin-coexpressing HEK293T cells were treated with SARS-S-CRD-WT peptide at indicated dose. Co-IP was performed to detect the interaction of S protein and Ezrin, S-CRD-Mut peptide taken as control

The evolutionary homology of Cys composition in CRD among different HCoVs initiated us to speculate the existence of a core Cys structure that determines the function of S protein. We first mutated 10 Cys in CRD of SARS-CoV-2 S protein into Ala, respectively. Results of cell fusion assay showed that a single C1241A (C4A) mutation, but not mutation at the other nine Cys, largely destroyed the syncytia formation (Fig. 1b and Supplementary Fig. S3a, b). However, the individual reversion of A1241C (A4C) failed to rescue the defect of syncytia formation induced by S-mCRD (Fig. 1b). Further reverse mutation found that the deficient syncytia formation induced by S-mCRD was only partially rescued when half of (that is 5) Ala residues adjacent to C1241 were reversed to Cys (A1/2/3/4/5C). Interestingly, adding an additional reverse mutation, such as A1/2/3/4/5/6C mutant, fully recovered S-mCRD-induced cell fusion and syncytia (Fig. 1b and Supplementary Fig. S3a, b). Consistently, both mCRD and C4A greatly reduced the pseudoviral infection of SARS-CoV-2, which was completely rescued by A1/2/3/4/5/6C reversion (Fig. 1b). These results suggest that one central Cys at 1241 site (C4) and about half of total Cys residues together constitute a functional CRD motif of SARS-CoV-2 S protein.

We then came to verify whether this functional CRD motif exists in other HCoVs. As shown in Supplementary Fig. S4a, b, single C1223A mutation (C4A) in SARS-CoV S-CRD domain with 9 Cys and the C1323A substitution (C3A) in MERS-CoV S-CRD with 7 Cys failed to cause syncytia production, while the syncytia formation defect induced by S-mCRD was completely rescued by A1/2/3/4/5/6C reversion in SARS-CoV and A1/2/3/4C recovery in MERS-CoV. This was further verified by pseudoviral infectivity assay (Supplementary Fig. S4c). Together, these data suggest that the functional S-CRD motif determining cell-cell fusion and viral infection in multiple HCoVs may follow a same pattern of composition, i.e., an indispensable core Cys and N adjacent Cys (*N* ≈ half the number of all Cys in CRD), named the 1 + *N* rule.

To address whether CRD works as an intervention target for pan-coronavirus infection, we synthesized three cell-penetrating peptide (CPP)-fused CRD peptides (S-CRD-WT) targeting five HCoVs respectively, peptides harboring C → A mutation (S-CRD-Mut) as negative controls (Fig. 1c). Cell fusion assay showed that treatment with SARS-S-CRD-WT peptide significantly inhibited the early cell fusion and later syncytia formation in a dose-dependent manner without significant cytotoxicity, while SARS-S-CRD-Mut peptide failed to do so (Supplementary Fig. S5a, b and Fig. 1d). Furthermore, results of pseudovirus infectivity assay found that SARS-S-CRD-WT peptide significantly inhibited SARS-CoV-2 infection (Fig. 1d). Similarly, S-CRD-WT but not S-CRD-Mut peptide at 20 μM significantly restricted S protein-induced cell fusion and pseudovirus entry of SARS-CoV, MERS-CoV, HCoV-229E and HCoV-NL63 (Supplementary Fig. S5c and Fig. 1e). These data suggest S-CRD peptide as an effective approach to inhibit cell fusion and viral infectivity of multiple HCoVs.

To assess the inhibitory activity of S-CRD peptide against authentic coronavirus infection, HCoV-229E and SARS-CoV-2 infection models were included. As shown in Fig. 1f, 229E-S-CRD-WT peptide significantly reduced HCoV-229E titer, cellular viral RNA, and N protein levels in a dose-dependent manner and the IC_50_ value on inhibiting HCoV-229E titer was about 1.3 μM. Treatment with 20 μM S-CRD-WT peptide led to a decrease of 3.0 log^10^ TCID_50_ in viral titer and a 100-fold decrease of viral RNA (Fig. 1f). Whereas cell viability was not affected by S-CRD peptide at 60 μM (Supplementary Fig. S6a) and the excellent selectivity index (SI, the ratio of CC_50_/IC_50_) exceeded 46.1. At 4 days post-infection (d.p.i), HCoV-229E caused almost all cell apoptosis (cytopathic effect, CPE) in the vehicle and S-CRD-Mut peptide control-treated cells, while treatment with ≥5 μM 229E-S-CRD-WT peptide completely prevented CPE production (Supplementary Fig. S6b). Even at 6 d.p.i., 5 μM 229E-S-CRD-WT peptide still prevented most cells from apoptosis in HCoV-229E infected cells (Supplementary Fig. S6c). To clarify which phase of viral infection that is targeted by 229E-S-CRD-WT peptide, a one-step growth curve and the time of addition assays were included. As shown in Supplementary Fig. S6d, HCoV-229E virions were released 12–18 h post-infection (h.p.i.) in Huh7 cells and S-CRD-WT peptide displayed the strongest antiviral effect in the process of viral entry and mainly inhibited the early stage of HCoV-229E infection.

Further, we evaluated the inhibition effect of S-CRD peptide on different variants of SARS-CoV-2. Expectedly, treatment with SARS-S-CRD-WT peptide significantly reduced the levels of SARS-CoV-2 alpha viral RNA in supernatant of Calu-3 cells with an IC_50_ value of about 4.8 μM (Fig. 1g), and no significant cytotoxicity was observed at 60 μM (Supplementary Fig. S6e). The SI was higher than 12.5. Strikingly, 20 μM S-CRD-WT peptide exhibited a comparable inhibitory activity as 20 μM remdesivir, inducing a 400-fold reduction in viral RNA copies, with an inhibition rate of 99.7% (Fig. 1g and Supplementary Fig. S6f). Notably, treatment with S-CRD-WT peptide also restricted the replication of SARS-CoV-2 omicron variant BA.5 in Calu-3 cells (Fig. 1h). Furthermore, S-CRD-WT peptide treatment not only reduced viral RNA but also greatly decreased N protein levels in SARS-CoV-2 alpha and omicron variants infected ACE2-A549 cells, while S-CRD-Mut showed no effects (Supplementary Fig. S6g). Collectively, these results illustrate that the S-CRD peptide is a potential broad-spectrum inhibitor of HCoVs infection.

S-mediated membrane fusion is a complex process that requires concerted action of receptor-binding, S protein cleavage, and subsequent fusion pore formation and expansion. However, subcellular components and immunofluorescence analysis did not detect the obvious difference in proteolytic processing and cellular localization between S-WT and S-mCRD (Supplementary Fig. S7a). It is well known that the reorganization of cellular actin affects the fusion pore and viral entry.^4^ As expected, treatment with cytochalasin B, a cell-permeable F-action formation inhibitor, completely blocked S-induced syncytia of SARS-CoV-2, SARS-CoV, and MERS-CoV (Fig. 1i). To seek the host protein coupling S-CRD with cytoskeleton remodeling, we performed co-immunoprecipitation (Co-IP) and mass spectrometry (MS) analysis to screen CRD-binding host factors during the cell–cell fusion process (Fig. 1j). Twenty-one proteins were identified in S-WT group but not in S-mCRD and vector controls, and only Ezrin involves in the regulation of actin cytoskeleton pathway (Fig. 1j). Co-IP assay further confirmed the specific binding of Ezrin with S-WT but not S-mCRD (Fig. 1k). Consistent with previous studies showing that Ezrin links actin cytoskeleton to cell membrane to regulate membrane behaviors,^5^ Ezrin silence strongly repressed the early cell fusion and later syncytia formation induced by SARS-CoV-2, SARS-CoV and MERS-CoV S proteins (Fig. 1l and Supplementary Fig. S7b, c). Specifically, pull-down assay found that Biotin-S-CRD-WT interacted with Ezrin while Biotin-S-CRD-Mut did not (Fig. 1m). In accordance, fluorescent colocalization analysis also confirmed the interaction of S-CRD-WT peptide with GFP-Ezrin (Supplementary Fig. S7d). Competitive assay showed that treatment with S-CRD-WT peptide inhibited the binding of S with Ezrin in a dose-dependent manner (Fig. 1n). All these data suggest that CRD interacts with Ezrin to initiate S-mediated membrane fusion.

Taken together, our study provides a novel strategy targeting CRD domain with peptide to efficiently against broad-spectrum HCoVs infection. Mechanistically, S-CRD peptide competitively binds to Ezrin with S protein to inhibit subsequent membrane fusion. Functional dissection of SARS-CoV-2 CRD domain found that Cys residue at 1241 site was palmitoylated and the mutation of 1240/1241 Cys led to a significant reduction in viral infectivity.^6^ However, the reversion of Cys at 1240/1241 alone failed to rescue the S-mCRD mediated defect of pseudovirus infectivity,^6^ suggesting a synergistic effect between the palmitoylated Cys and other Cys sites in controlling CRD biological function, which is consistent with our results. Strikingly, these S-CRD-WT peptides showed cross-inhibitory activity. As shown in Supplementary Fig. S8, HCoV-229E replication was also significantly inhibited by SARS-S-CRD-WT and MERS-S-CRD-WT peptides, suggesting the broad-spectrum cross-inhibitory activity of S-CRD peptide. Further efforts are required to optimize this antiviral peptide by in silico design and modification to improve its pharmaceutical property against broad-spectrum HCoVs infection.

## Supplementary information

supplemental materials

## Data Availability

Further information and requests for resources and reagents should be directed to and will be fulfilled by the Lead Contact C.M. (machunhong@sdu.edu.cn).

## References

[1] Kuhlmann, C. et al. Breakthrough infections with SARS-CoV-2 omicron despite mRNA vaccine booster dose. *Lancet* **399**, 625–626 (2022); erratum **399**, 628 (2022). 10.1016/S0140-6736(22)00090-3 PMC8765759 35063123

[2] Xia S (2020). Inhibition of SARS-CoV-2 (previously 2019-nCoV) infection by a highly potent pan-coronavirus fusion inhibitor targeting its spike protein that harbors a high capacity to mediate membrane fusion. Cell Res..

[3] Wu Z (2021). Palmitoylation of SARS-CoV-2 S protein is essential for viral infectivity. Signal Transduct. Target. Ther..

[4] Taylor MP, Koyuncu OO, Enquist LW (2011). Subversion of the actin cytoskeleton during viral infection. Nat. Rev. Microbiol..

[5] Clucas J, Valderrama F (2015). ERM proteins in cancer progression. J. Cell Sci..

[6] Puthenveetil R (2021). S-acylation of SARS-CoV-2 spike protein: mechanistic dissection, in vitro reconstitution and role in viral infectivity. J. Biol. Chem..

