# Supplementary material for: Peptide targeting the interaction of S protein cysteine-rich domain with Ezrin restricts pan-coronavirus infection
Source: Signal Transduct Target Ther. 2023 Jan 18;8:19. doi: 10.1038/s41392-022-01244-z (PMC9845329; doi:10.1038/s41392-022-01244-z)
Supplement: Supplementary file 1 — supplemental materials [file 41392_2022_1244_MOESM1_ESM.doc]

Supplementary Materials for

**Peptide targeting the interaction of S protein cysteine-rich domain with Ezrin restricts pan-coronavirus infection**

Zhuanchang Wu^1,†^, Xiaobo Lei^2,†^, Xin Wang^3,†^, Zhaoying Zhang^1^, Yuming Li^4^, Lifen Gao^1^, Xiaohong Liang^1^, Peihui Wang^5^, Jianwei Wang^2,*^ and Chunhong Ma^1,*^

Correspondence to Chunhong Ma ([machunhong@sdu.edu.cn) and](mailto:machunhong@sdu.edu.cn) and) Jianwei Wang ([wangjw28@163.com](mailto:wangjw28@163.com))

**This PDF file includes:**

Materials and Methods

Supplementary figures S1-S8

**Materials and Methods**

***Cells, peptides, viruses and plasmids***

The human embryonic kidney cell line 293T (HEK293T) and the human hepatoma cell line Huh7 were purchased from the Shanghai Cell Collection (Shanghai Institutes for Biological Sciences, Chinese Academy of Sciences, Shanghai, China). HEK293T-ACE2 cell line was established to stably express human ACE2 receptor. All cell lines were cultured in Dulbecco’s Modified Eagle’s Medium (DMEM) with 10 % fetal bovine serum (FBS). Transfections of HEK293T were performed using Lipofectamine 2000 (Thermo Fisher Scientific, Waltham, USA) according to the manufacturer’s protocol. All peptides were synthesized by Genscript Co., Ltd (Nanjing, China) with a purity of > 95 % (tested by high-performance liquid chromatography). Cytochalasin B was purchased from Med Chem Express (HY-16928, MCE, USA). The commercial antibodies used for western blot were: SARS-CoV-2 nucleocapsid antibody (40143-MM08, Sino Biological Inc., Beijing, China), HCoV-229E nucleocapsid antibody (40640-T62, Sino Biological Inc.), Ezrin antibody (ab40839, Abcam, USA), β-actin monoclonal antibody (66009-I-Ig; Proteintech Group Inc., Rosemont, USA), anti–glyceraldehyde 3-phosphate dehydrogenase (GAPDH) antibody (66004-I-Ig; Proteintech Group Inc.), anti-Flag monoclonal antibody (M185-3L, MBL, Nagoya, Japan), and anti-HA monoclonal antibody (M180-3, MBL). The siRNA oligos against human Ezrin were synthesized from GenePharm (Shanghai, China) and their sequences were as follows: siEzrin-1#: 5’-CACCGUGGGAUGCUCAAAGTT-3’, siEzrin-2#: 5’-AAGGAAUCCUUAGCGAUGAGA-3’.

The pCAG-SARS-CoV-2-S and pCAG-SARS-CoV-S plasmids were preserved in our laboratory. The pcDNA3.1-MERS-S, pcDNA3.1-HCoV-229E-S and pcDNA3.1-HCoV-NL63-S expressed plasmids and HCoV-229E virus were kindly gifted by professor Cunjin Zhao in Guanzhou Medical University, China. The SARS-CoV-2 BJ (Beijing) strain of alpha variant and BA.5 strain of omicron variant in this study were isolated by the lab of professor Jianwei Wang. Plasmid expressing HA-tagged human Ezrin (HA-Ezrin) was generated by inserting a full-length coding sequence into the pCAGGS-HA vector. The Flag-S-CRD mutants and HA-Ezrin mutants were generated by KOD-Plus-Mutagenesis Kit (TOYOBO, Osaka, Japan).

***Coronavirus phylogenetic analysis***

Phylogenetic trees were constructed using the MEGA6.06. Accession numbers used for phylogenetic analysis are as follows: HCoV-229E (AOG74783.1), HCoV-NL63 (AKT07952.1), TGEV (NP_058424.1), PRCV (ABG89317.1), FcoV (ABI14448.1), PEDV (ALS35469.1), BcoV-1 (QTF66157.1), MHV (NP_045300.1), HCoV-OC43 (AMK59677.1), HCoV-HKU1 (AYN64561.1), BtCoV-Rm1 (ABD75332.1), BatSL-CoV-Rs3367 (AGZ48818.1), BatSL-CoV-WIV1 (AGZ48828.1), SL-CoV-WIV16 (ALK02457.1), SARS-CoV (YP_009825051.1), SARS-CoV-2 (YP_009724390.1), Pi-BatCoV-HKU5 (AWH65932.1), Ty-BatCoV-HKU4 (AWH65899.1), MERS-CoV (YP_009047204.1), Ro-BatCoV-HKU9 (YP_001039971.1), PDCoV (AQS99154.1), PorCoV-HKU15 (BAY00730.1), BuCoV-HKU11 (YP_002308479.1), CMCoV-HKU21 (AFD29244.1), ThCoV-HKU12 (YP_002308497.1) and IBV(CAC39300.1). The accession numbers used for S protein sequence alignment of *Coronavirinae* and *Toroviridae* are as follows: Bovine torovirus (QJT73572.1), Porcine torovirus (BBK20260.1), White bream virus (NC_008516.1). All S protein sequences of 43 SARS-CoV-2 strains were extracted from the GISAID database (https://www.gisaid.org/).

***Cell-cell fusion assays***

S protein-expressing plasmid and pEGFP-N1 at a 4:1 ratio were cotransfected into HEK293T effector cells for 36 h. When assessing the inhibitory role of S-CRD peptide on S protein-mediated cell-cell fusion, at 12 h post-transfection (h.p.i), effector cells were treated with peptide at the indicated concentration for another 24 h. Huh7 cells naturally expressing angiotensin-converting enzyme 2 (ACE2), dipeptidyl peptidase 4 (DPP4), and aminopeptidase N (APN) receptors and HEK293T-ACE2 were used as target cells. 4 × 10^4^ target cells were incubated in 24-well plates at 37 ℃ for 4 h, followed by the addition of 20 × 10^4^ HEK293T effector cells were cocultured. For SARS-CoV and NL63 S-mediated cell-cell fusion, it needed to add trypsin (80 ng/ml) in DMEM without FBS to promote cell fusion. After coculture at indicated times, fused cells and syncytium formation were observed under an inverted fluorescence microscope. After coculture for 24 h and 48 h, syncytium formation was observed under the inverted fluorescence microscope. Four fields in each well were randomly selected for counting the fused cells. The fused cells are at least twice as large as the unfused cells, and the fluorescence intensity in the fused cell became weak due to the diffusion of enhanced green fluorescent protein (EGFP) from one effector cell to the target cells. The cell-cell fusion rate was calculated relative to the level at vehicle control (set as 100) [(number of the fused cells in peptide-treated groups/number of the fused cells in a vehicle control) × 100 %].

***Production and infection of HCoVs S pseudoviruses***

The HCoVs S pseudotyped HIV-1 single-round luciferase virus were produced in HEK293T cells as previously described[^1^](#_ENREF_1). In brief, PLP1, PLP2, pCDH-CMV-Luciferase-CopGFP, and different HCoVs S expressing plasmids were cotransfected into HEK293T cells and the supernatant containing pseudoviruses were quantitated by detecting viral RNA copies. When assessing the role of S-CRD peptide in regulating viral infectivity, at 12 h post-transfection, S-CRD peptide was added into medium at indicated doses for another 36 h and then harvested pseudoviruses to quantitate. To measure viral infectivity, Huh7 cells seeded into 48-well plates were infected with pseudotyped viruses at equal RNA copies supplemented with polybrene (4 mg/ml) and then lysed after 72 h post-infection with passive lysis buffer (Promega, Madison, WI, USA) to measure firefly luciferase activity using a luciferase reporter assay system (Promega) according to the manufacturer’s protocol. The viral infection rate was calculated by analyzing the mean firefly luciferase activity as to the control group (set as 100 %).

***Inhibition of authentic HCoVs replication***

The inhibitory activity of peptide against HCoV-229E and SARS-CoV-2 replication was assessed in Huh7 cells and Calu-3 cells, respectively. Briefly, cells at approximately 70-80 % confluence were washed with PBS and inoculated with HCoV-229E at a multiplicity of infection (MOI) of 0.1 or SARS-CoV-2 at an MOI of 0.01 for the indicated times, then the inocula were removed and the cells were maintained in medium containing 2 % FBS at 37 ˚C. 36 h later, HCoV-229E level in supernatant was titrated by TCID_50_ assay in Huh7 cells based on the cytopathic effect (CPE); HcoV-229E N protein and viral RNA and levels were detected by western blot and RT-qPCR using 229E-F: 5’-TGAAGATGCTTGTACTGTGGCT-3’ and 229E-R: 5’-CTGTCATGTTGCTCATGGGG-3’; SARS-CoV-2 RNA levels in the supernatant were quantitated by RT-qPCR as previously described[^2^](#_ENREF_2). 4-6 days post-infection (d.p.i), HCoV-229E-induced CPE was observed under a light microscope using the bright field.

***Mass-spectrometry analysis***

2 × 10^7^ HEK293T cells were transfected with Flag-S, Flag-S-mCRD, and empty vector for 36 h and subsequently cocultured with 2 × 10^7^ HEK293T-ACE2 cells for 4 h to induce cell fusion. Then cells were collected to the lysate and incubated with anti-Flag antibody for 5 h and Protein-A/G beads for another 1 h at 4 °C under constant agitation. After careful wash with PBST four times, the beads were boiled with 25 μl 1 × SDS loading buffer and protein samples were then sent to BGI Co. Ltd. (Shenzhen, China) for LC-MS/MS analysis. The candidates with ≥ 15 % coverage rate and ≥ 5 unique peptides were used for Venn analysis.

***Cytotoxicity assay***

Cytotoxicity of the peptide to the cells (Huh-7, HEK293T, and Calu-3 cells) was tested by using the Cell Counting Kit-8 (CCK-8; Dojindo, Kumamoto, Japan). Briefly, each cell type was seeded into the wells of a 96-well microtiter plate (10^4^ per well) and incubated at 37 °C for 36 h with DMED containing S-CRD peptide at graded concentrations. Then CCK-8 solution was added, followed by an additional incubation for 30 min, the absorbance was measured at 450 nm wavelength with a reference wavelength of 630 nm.

***Statistical analysis***

All statistical analyses were performed using the Prism software package version 8 (GraphPad software). Unpaired Student t-test and one-way ANOVA were performed to determine statistical significance between groups. Significance levels are indicated by asterisks: **P* < 0.05; ***P* < 0.01.

**Supplementary Figures**


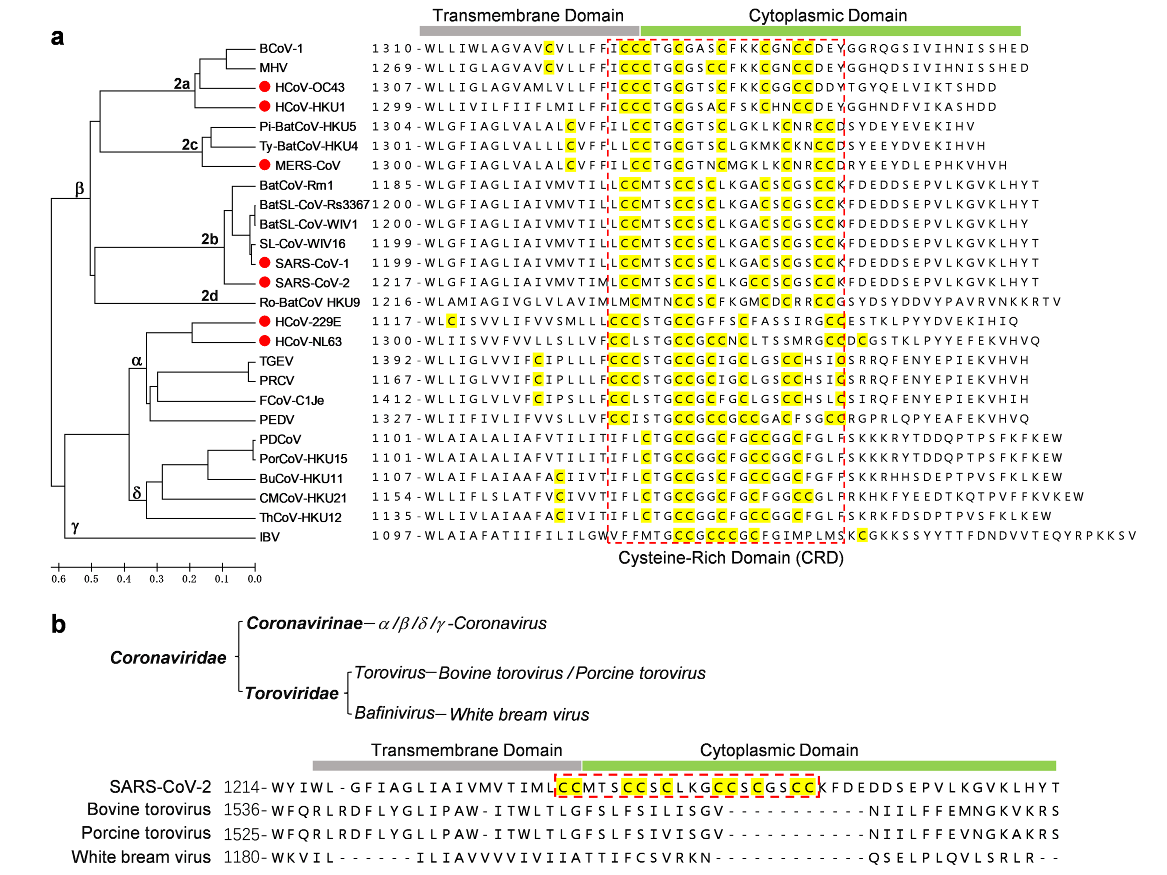


**Supplementary Fig. S1 The sequence analysis of CRD domain in coronavirus S proteins. a**, The phylogenetic trees were constructed using the MEGA6.06 basing S protein sequences of representative strains of *Coronavirinae* subfamily and the transmembrane and cytoplasmic domain of S protein were aligned. **b**, The sequence alignment of the intracellular domain of S protein from different representative strains of *Coronavirinae* and *Toroviridae*. Red box indicates the CRD domain and cysteines are labeled with yellow shadows.


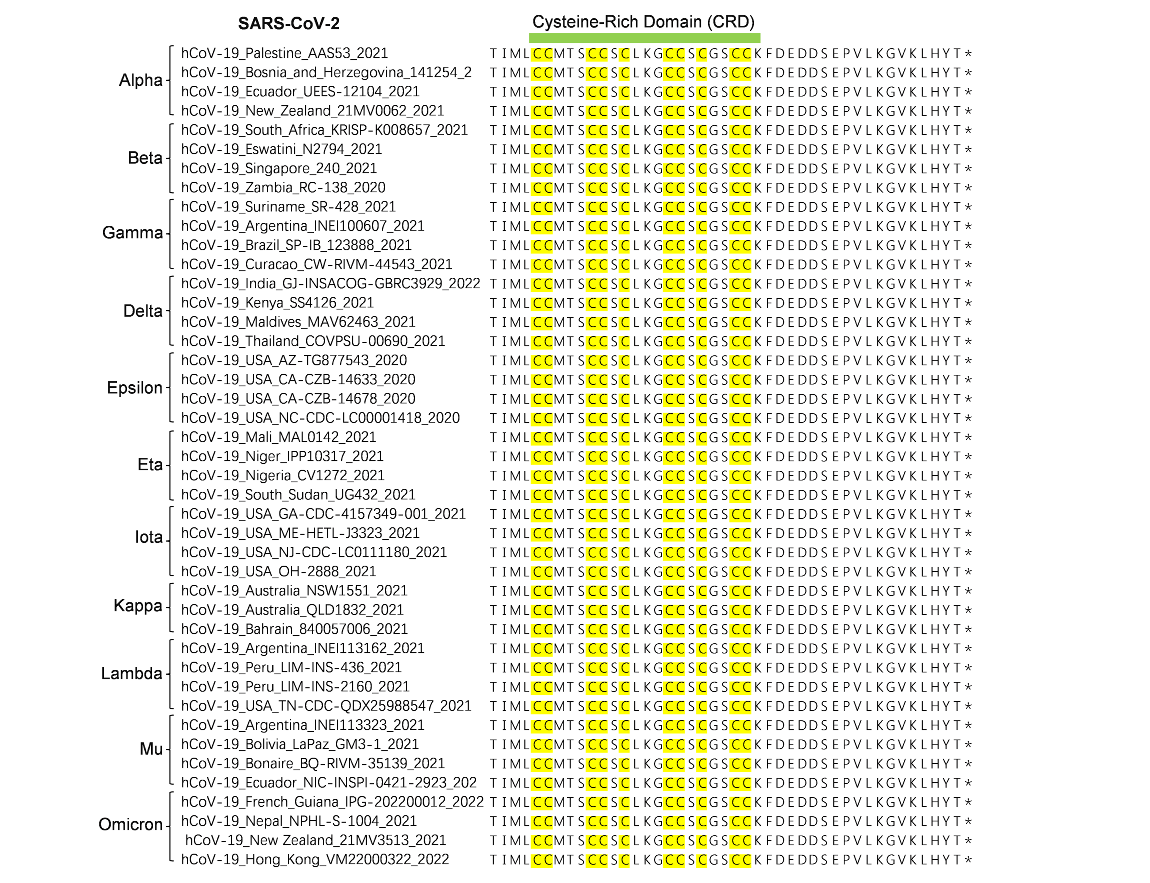


**Supplementary Fig. S2 The sequence alignment of CRD domain from different SARS-CoV-2 strains.** The sequence alignment of the intracellular domain of S protein from different SARS-CoV-2 strains. Cysteines are labeled with yellow shadows.


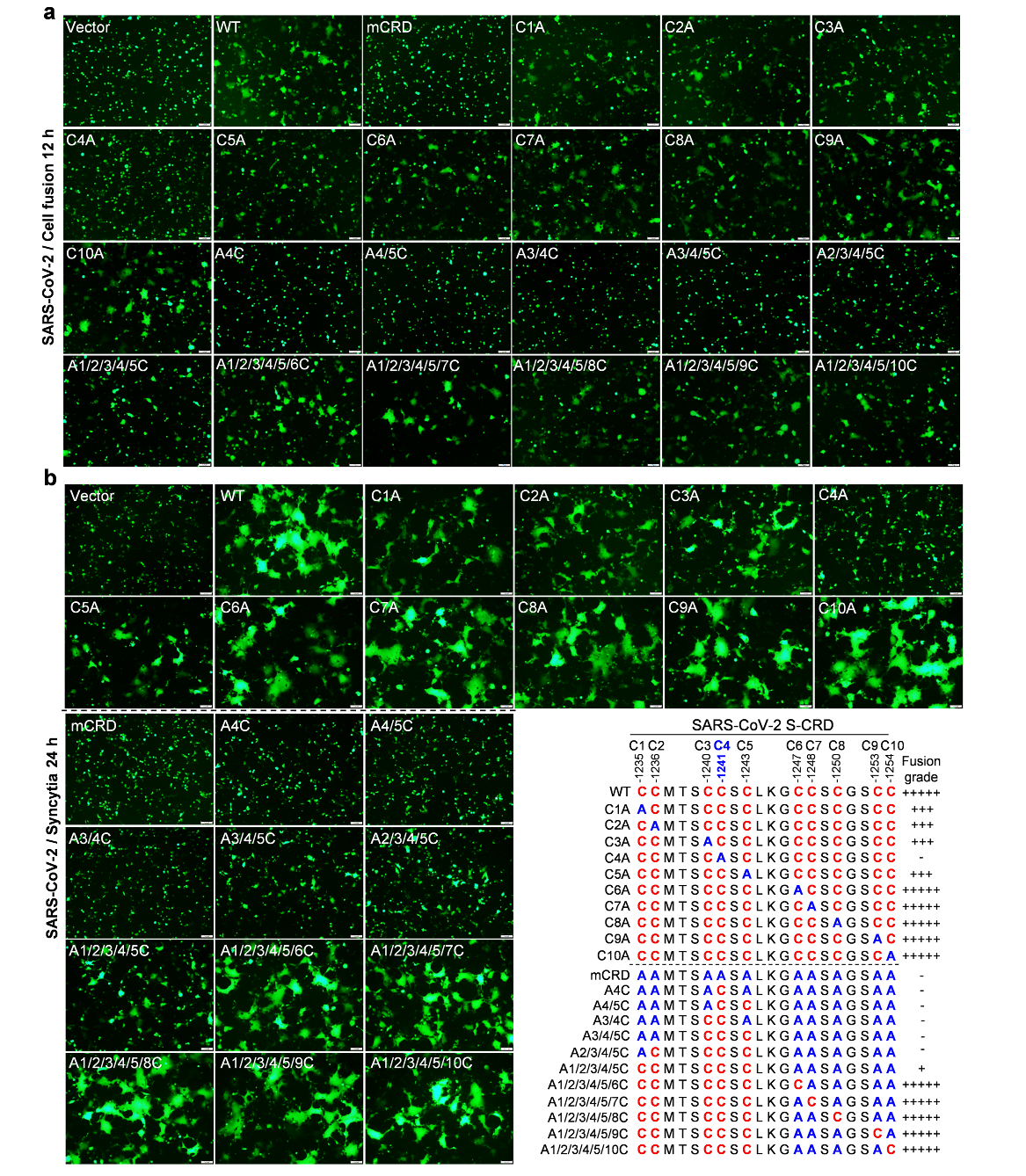


**Supplementary Fig. S3 Critical Cys residues that determine SARS-CoV-2 S-CRD-mediated cell fusion.** HEK293T cells transfected with SARS-CoV-2 S-WT/GFP or a series of CRD mutants/GFP were cocultured with Huh7 cells for 12 h (**a**) and 24 h (**b**), early cell fusion and later syncytia formation were visualized by fluorescent imaging. The scale bar indicates 100 µm.


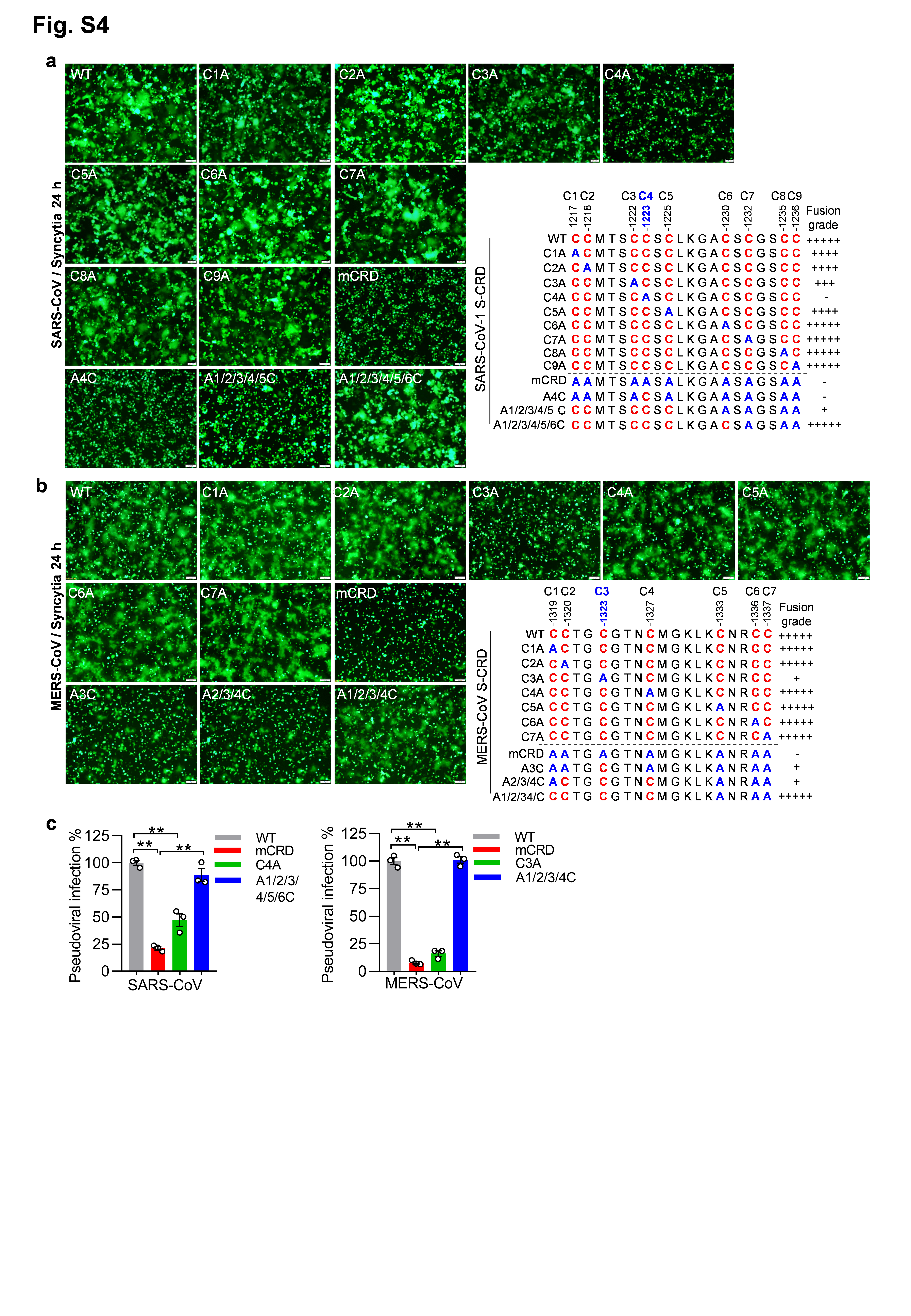


**Supplementary Fig. S4 Functional CRD motif that determines SARS-CoV and MERS-CoV S protein-mediated cell fusion and pseudoviruses infection. a-b,** HEK293T cells transfected with S-WT/GFP or a series of CRD mutants/GFP of SARS-CoV (**a**) and MERS-CoV (**b**) were cocultured with Huh7 cells for 24 h, syncytia formation was visualized by fluorescent imaging. The scale bar indicates 100 µm. **c,** Pseudoviruses containing S-WT or CRD mutants of SARS-CoV and MERS-CoV infected Huh7 cells for 72 h, the firefly luciferase activity was detected to analyze viral infectivity (*n*=3). One-way ANOVA, **P*<0.05; ***P*<0.01.


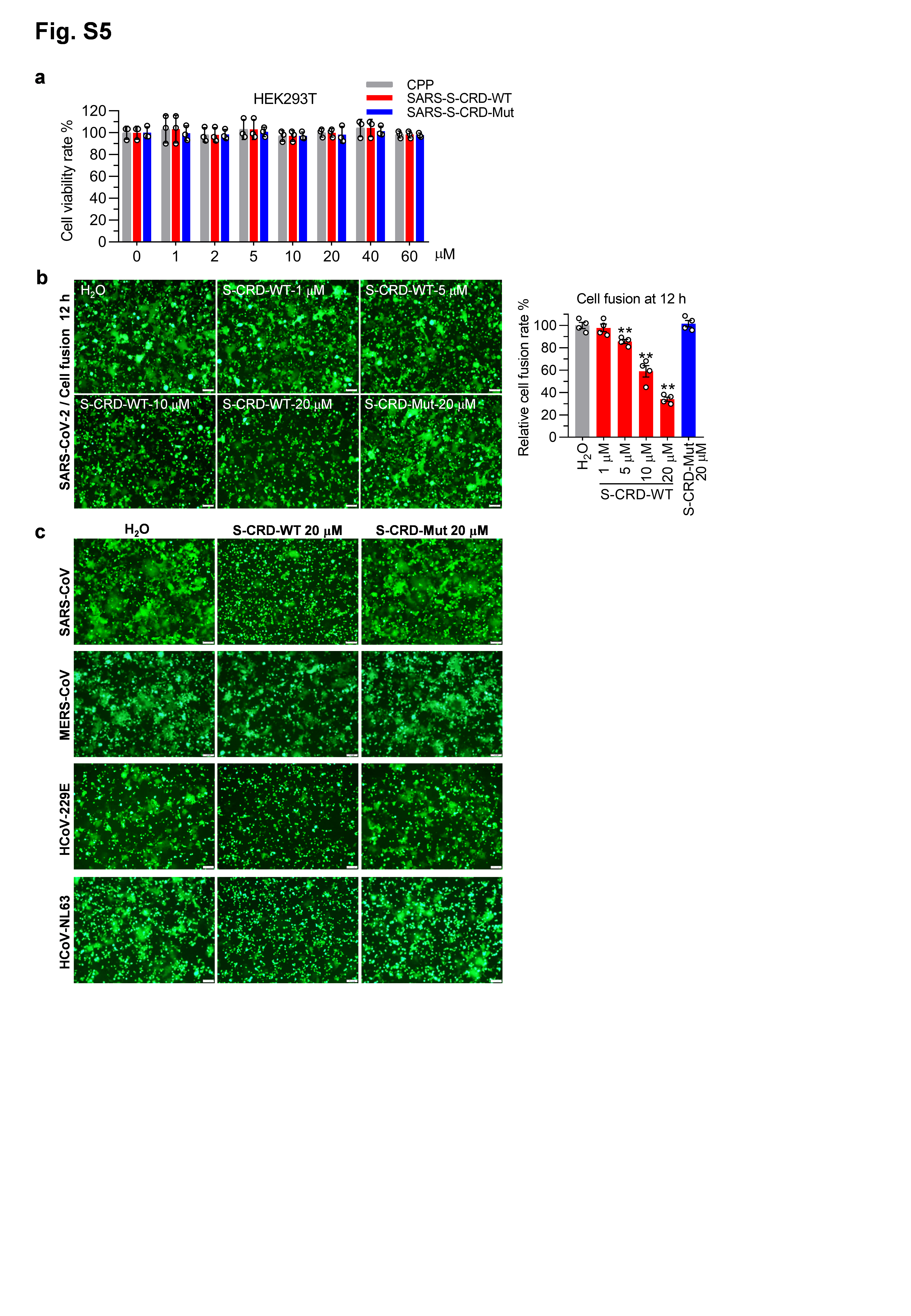


**Supplementary Fig. S5 S-CRD peptide inhibits S protein-induced cell fusion and viral infectivity of multiple HCoVs. a**, HEK293T cells were treated with CPP, SARS-S-CRD-WT, or SARS-S-CRD-Mut peptides at the indicated concentrations for 24 h, and the cytotoxicity was analyzed by CCK-8 assay (*n*=3). **b**, SARS-CoV-2 S-WT/GFP-coexpressed HEK293T cells were treated with S-CRD peptide at indicated doses and cocultured with Huh7 cells for 12 h, and cell fusion was visualized by fluorescent imaging. The scale bar indicates 100 µm. **c**, Syncytia formation mediated by S protein of SARS-CoV, MERS-CoV, HCoV-229E, and HCoV-NL63 was inhibited by S-CRD peptide (*n*=3). The scale bar indicates 100 µm. One-way ANOVA, **P*<0.05; ***P*<0.01.


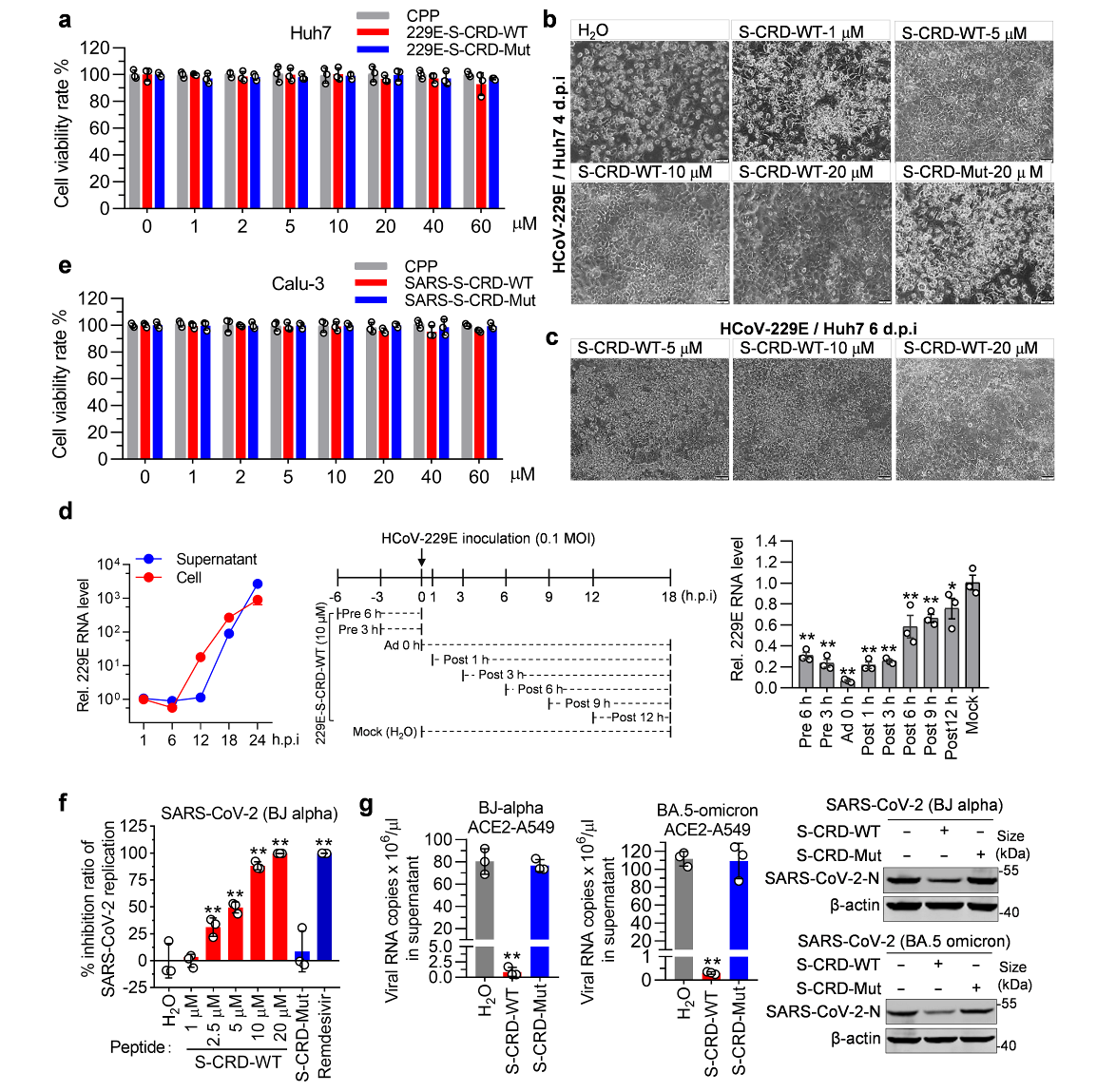


**Supplementary Fig. S6 S-CRD peptide represses replication of HCoV-229E and SARS-CoV-2. a**, Huh7 cells were treated with CPP, 229E-S-CRD-WT and 229E-S-CRD-Mut peptides at the indicated concentrations for 36 h, and the cytotoxicity was analyzed by CCK-8 assay (*n*=3). **b-c**, S-CRD peptide inhibits HCoV-229E induced cytopathic effect (CPE). Huh7 cells infected with HCoV-229E virus at 0.1 MOI were treated with 229E-S-CRD peptide. CPE was observed with light microscope at 4 d.p.i (**b**) and 6 d.p.i (**c**). The scale bar indicates 50 µm. **d**, HCoV-229E virus infected Huh7 cells at 0.1 MOI, and viral RNA levels in cell and supernatant were detected by RT-PCR to map the one-step growth curve of HCoV-229E virus (left panel). HCoV-229E-infected Huh7 cells were treated with 10 µM 229E-S-CRD-WT at the indicated times, and viral RNA levels were determined 18 h.p.i by RT-PCR (right panel). **e**, Calu-3 cells were treated with CPP, SARS-S-CRD-WT, or 229E-S-CRD-Mut peptides at the indicated concentrations for 36 h, and the cytotoxicity was analyzed by CCK-8 assay (*n*=3). **f**, S-CRD peptide inhibits the replication of alpha variant of SARS-CoV-2. 0.01 MOI SARS-CoV-2 (BJ strain) infected Calu-3 cells for 2 h and then treated with SARS-CoV-2-S-CRD peptide at indicated doses for 36 h, viral copies in the supernatant were measured with RT-qPCR (*n*=3) and % inhibition rate was calculated relative to the vehicle control (set as 0). **g**, S-CRD peptide inhibits the replication of different SARS-CoV-2 variants. 0.01 MOI SARS-CoV-2 infected ACE2-A549 cells for 2 h and then treated with 20 µM SARS-S-CRD peptide for 36 h, viral copies in the supernatant were measured with RT-qPCR (*n*=3). One-way ANOVA, **P*<0.05; ***P*<0.01.


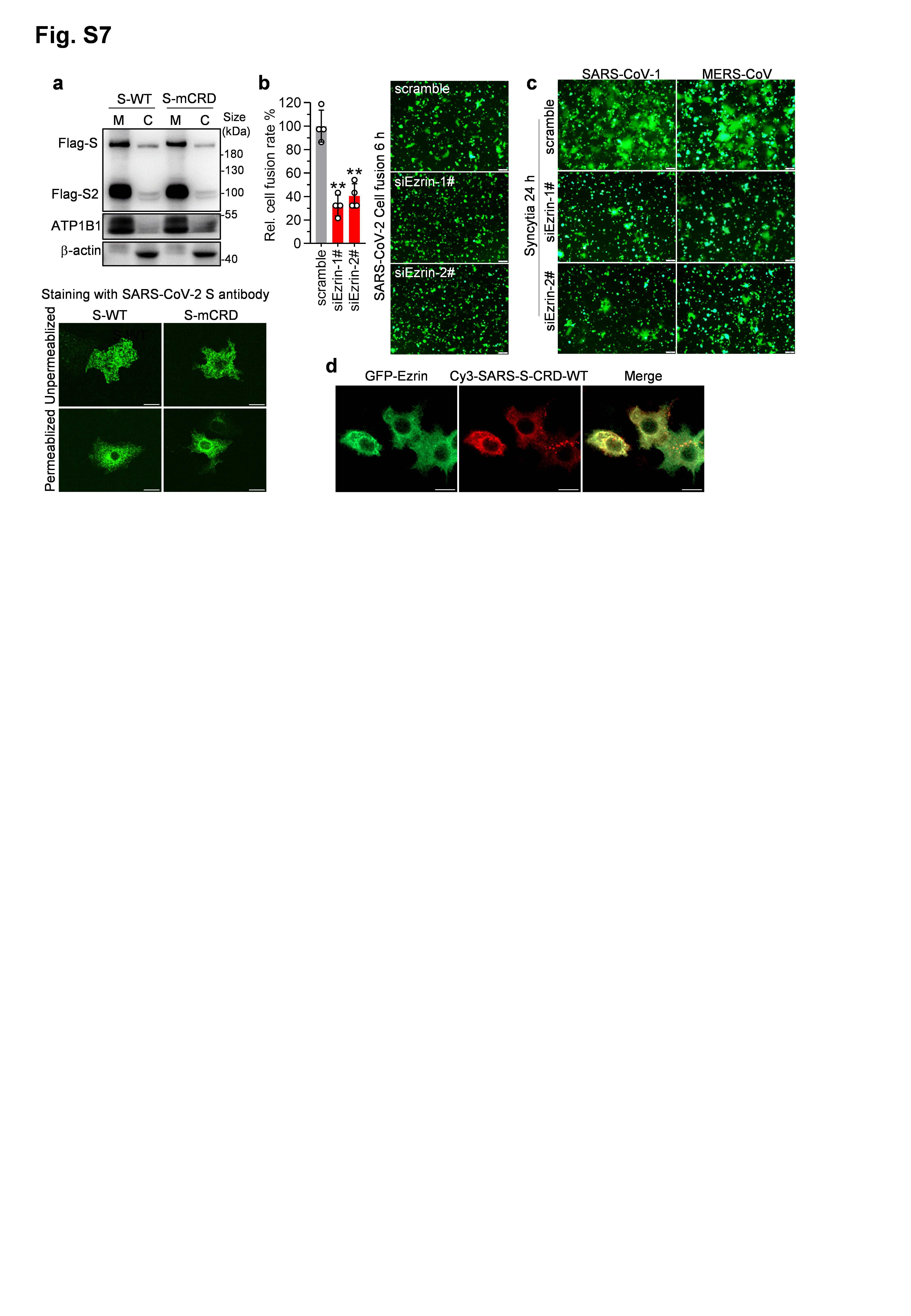


**Supplementary Fig. S7 Ezrin is essential for S-mediated membrane fusion. a**, SARS-CoV-2 S-WT and S-mCRD were overexpressed in HEK293T for 48 h. The membrane and cytoplasm distribution of S protein was detected by western blot (upper panel) and IFA (lower panel). The scale bar indicates 20 µm. **b**-**c**, HEK293T cells were transfected with Ezrin-siRNA for 36 h and S-WT/GFP for another 36 h, cell fusion was performed to analyze early cell fusion induced by SARS-CoV-2 S protein post 6 h coculture (**b**) or late syncytia formation induced by MERS-CoV and SARS-CoV S proteins post 24 h coculture (**c**). The scale bar indicates 100 µm. **d**, Huh7 cells transfected with GFP-Ezrin were incubated with biotin-SARS-S-CRD peptide and stained with Cy3-streptavidin, the fluorescence colocalization was analyzed by confocal microscopy. The scale bar indicates 20 µm.


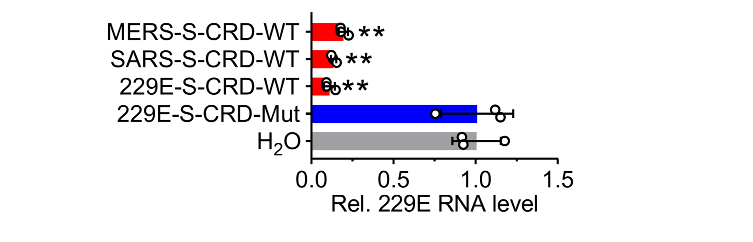


**Supplementary Fig. S8 The cross-inhibitory activity of S-CRD peptides on HCoV-229E replication.** Huh7 cells were infected with HCoV-229E virus at 0.1 MOI for 6 h and then treated with 10 µM 229E-S-CRD, SARS-S-CRD, and MERS-S-CRD peptides for 36 h, respectively. Viral RNA levels were detected by RT-qPCR (*n*=3).

**References**

1 Wu, Z. *et al.* Palmitoylation of SARS-CoV-2 S protein is essential for viral infectivity. *Signal Transduct Target Ther*. **6**, 231, (2021).

2 Zhou, Z. *et al.* Sensing of cytoplasmic chromatin by cGAS activates innate immune response in SARS-CoV-2 infection. *Signal Transduct Target Ther*. **6**, 382, (2021).
